# Polymer M-Z Thermal Optical Switch at 532-nm Based on Wet Etching and UV-Writing Waveguide

**DOI:** 10.3390/polym11060995

**Published:** 2019-06-04

**Authors:** Jiawen Lv, Yue Cao, Baizhu Lin, Yue Yang, Yue Sun, Shuai Li, Yunji Yi, Fei Wang, Daming Zhang

**Affiliations:** State Key Laboratory of Integrated Optoelectronics, College of Electronic Science & Engineering, Jilin University, Changchun 130012, China; lvjw18@mails.jlu.edu.cn (J.L.); yuecao17@mail.jlu.edu.cn (Y.C.); linbz17@mails.jlu.edu.cn (B.L.); a2604702999@163.com (Y.Y.); sun16@mails.jlu.edu.cn (Y.S.); 17607178920@163.com (S.L.); wang_fei@jlu.edu.cn (F.W.); zhangdm@jlu.edu.cn (D.Z.)

**Keywords:** polymer waveguide, thermal optical effect, UV-writing, optical switch

## Abstract

Polymer thermal optical switches have low power consumption and 532 nm is the communication window of polymer fiber. Polymer thermal optical switches at 532 nm are rarely reported, because of switching extinction ratio properties that are restricted by modes of the waveguide. Single mode waveguide at 532 nm is hard to fabricate due to the dissolution of core and cladding materials. A polymer M-Z thermal optical switch at 532 nm was first demonstrated based on the wet etching method. The proposed thermal optical switch was consisted of silica substrate, photosensitive polymer core, and cladding material. The device was fabricated and tested with the power consumption of 6.55mW, extinction of 4.8 dB, and switching time of 0.23 ms (rise)/0.28 ms (down). An optimized switch structure combining with the UV-writing technique and graphene thermal conduction layer was proposed based on the experiments above. A side electrode was designed to reduce the power consumption and the switching time. The optimized device was calculated to have a power consumption of 1.5 mW. The switching time of the UV-writing device was simulated to be 18.2 μs (rise) and 85 μs (down). The device is promising in the wearable device and laser radar area.

## 1. Introduction

As a new method of communication, optical signal achieves faster network speed and wider bandwidth. Optical switch is an important device for optoelectronic and integrated optics. It plays the role of protecting, testing, monitoring, and management of the network. The thermal optical (TO) switch, as the important part of the optical switch, acts a pivotal part in showing great promise in low-speed fiber-optic communication systems [1,2,3]. At present, the wavelengths of the reported thermal optical switches are mostly at 1550 nm and 650 nm, and the thermal optical switch working at 650 nm is mainly used in short-range communication system [4,5,6]. In recent years, 532 nm wavelength has gained wide attention in areas of industrial control, photobiology, and short-range communication. Corresponding lasers and detectors have been widely used. The corresponding optical signal processing devices art the chip level are progressing. In particular, optical switches in the photonic integrated circuit at 532 nm wavelength are promising in optical computing chip, wearable photobiology chip, and underwater laser radar [7,8].

Polymer materials have a high thermal optical coefficient, which can significantly reduce power consumption of thermal optical devices. At present, the polymer waveguides mainly include all-polymer devices and hybrid polymer/silica devices. In 2006, Young-Ouk Noh reported a thermal optical of polymer waveguide, and the switching crosstalk is reduced to −70 dB with an applied electrical power of 200 mW [9]. In 2013, Changming Chen reported an all-polymer thermal optical waveguide switch arrays based on novel grafting poly (methyl methacrylate) (PMMA) materials. The M-Z interferometer thermal optical switch on–off time is 600 μs. The extinction ratio is about 8.2 dB and the applied electric power as the switching power is about 22.6 mW at 650 nm. The all-polymer thermal optical switch has the advantage of low power consumption, but the response time is longer [10]. In 2012, Lei Liang fabricated a Mach–Zehnder interferometer (MZI) thermal optical switch at 1550 nm wavelength while using a hybrid silica/polymer waveguide structure. The switching power is 13 mW and the extinction ratio is 18.3 dB with the rise/fall time of 73.5/96.5 μs, respectively [11]. The hybrid silica/polymer structure ensures low power consumption and reduces the switching time. Therefore, the organic-inorganic hybrid integrated waveguide structure was selected in this paper.

The thermal optical switch can be divided into Mach-Zehnder (M-Z) thermal optical switch, multimode-interference (MMI) thermal optical switch, X-band thermal optical switch, and digital thermal optical switch [12,13,14]. M-Z optical switch has large process tolerance and lower power consumption. With the M-Z structure, the waveguide mode will have an effect on the extinction ratio and other parameters of the device. Therefore, it is crucial to achieve a single mode waveguide. For polymer materials, ridge the waveguide structure is an effective method to achieve single mode waveguide [15,16]. However, the single mode ridge polymer waveguide at 532 nm has large coupling loss between the device and the fiber. Another method is adjusting the refractive index of polymers. Due to the spin coating fabrication process, the waveguides with close refractive index difference are usually suffering from the core-cladding solution problem. Therefore, the high-quality single-mode waveguide is the key factor to the 532 nm M-Z thermal optical switch.

In this paper, we firstly designed and fabricated a M-Z thermal optical switch at 532 nm with a top electrode using the wet etching method. We then proposed a UV-writing method [17,18,19] to achieve few modes waveguide in order to optimize the extinction ratio. The side electrode structure without cladding was introduced to guarantee fast switching properties. The graphene thermal conduction layer would insure the high heating efficiency. The device parameters and performance were analyzed.

## 2. Materials and Methods

### 2.1. SU-8/PMMA Wet Etching M-Z Optical Switch with Top Electrode

**Figure 1:**
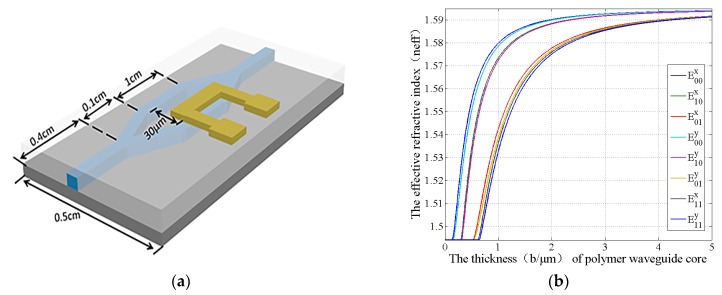
(**a**) The schematic of polymer/silica hybrid thermal optical switch. (**b**) The single mode condition of the polymer waveguide at 532 nm (The aspect ratio is 0.333).

Figure 1a shows the device structure. SU-8 is selected as the polymer waveguide core material due to its easy fabrication method. Its refractive index and thermal conductivity are 1.595 and 0.2 W/(m·K), respectively. Silicon is selected as the substrate and heat sink with 5 μm silica as the lower cladding. The refractive index and thermal conductivity of silica are 1.461 and 1.4 W/(m·K), respectively. PMMA is selected as the polymer upper cladding material, and its refractive index and thermal conductivity are 1.494 and 0.19 W/(m·K), respectively. The M-Z structure parameters are shown in Figure 1a with the branch width of 30 μm, the branch length of 1 cm, and the Y branch length of 0.1 cm. A single mode waveguide at 532 nm wavelength needs a waveguide dimension of 0.3 μm × 0.9 μm, according to Figure 1b. This dimension cannot be fabricated by lithography. In addition, it will increase the coupling loss. Therefore, the waveguide dimension is selected to be 3 µm × 9 µm.

**Figure 2:**
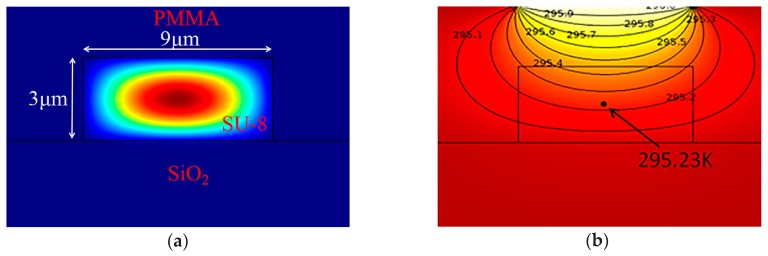
(**a**) The optical field of the fundamental mode in hybrid waveguide at 532 nm (**b**) the thermal field of the thermal optical switch.

Comsol Multiphysics and Matlab simulated the optical field and thermal field. Figure 2a is the optical field profile of the fundamental mode, Figure 2b shows the thermal field. The rise time and fall time of the device was simulated to be 218.55 μs and 372.79 μs, respectively. However, fiber coupling will excite all of the modes at the same time in the waveguide. Subsequently, the device cannot provide a meaningful low crosstalk.

**Figure 3:**
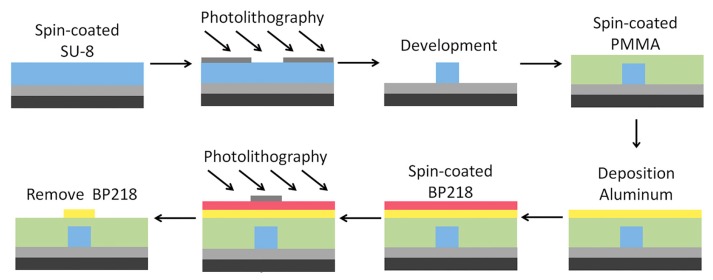
Process flow for fabricating the thermal optical switch.

We fabricated the device by the wet etching method. Figure 3 shows the device fabrication process. A 2.5 μm SiO_2_ layer was deposited on the silicon substrate as the under-cladding by the chemical vapor deposition (CVD) technique. A 3 μm thickness SU-8-2005 was fabricated on the SiO_2_ layer by spin-coater (WS-650MZ-23NPPB). After baking, ultraviolet (UV) photo-etching (ABM/6/350) using a waveguide mask was performed for 4 s (20 mW/cm^2^), and then the post bake and developing was performed. A 4 μm PMMA upper cladding layer was spun-coated and baked at 150 °C for 10 min with hot plate. Next, a 100 nm thick layer of aluminum was vaporized on the surface of the PMMA whileusing a coating machine (DM-300B), and the aluminum electrode was obtained by the BP218 mask wet etching method.

**Figure 4:**
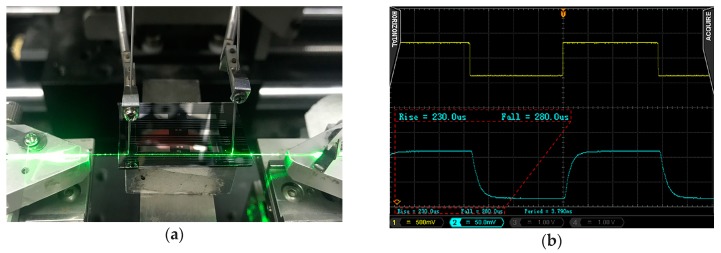
(**a**) The photograph of the device and its test system; and, (**b**) Switching response of the thermal optical switch.

The prepared device was tested at the 532nm wavelength. Figure 4a shows the photograph of the test system. Figure 4b shows the measured result. The rise time and fall time are 230 μs and 280 μs. The extinction ratio is 4.8 dB and the corresponding voltage change value is 2.4 V. The power consumption is 6.55 mW, according to the resistance of the heating electrode (0.88 kΩ).

The results indicate that the theoretical simulations accord with the test results. The switching time is faster than all polymer switches due to the high thermal conductivity of SiO_2_ lower cladding and silicon substrate. In addition, the device has lower power consumption than other polymer thermo-optic switches with the same structure due to its short wavelength. However, the extinction ratio of the device is relatively low. For the following discussion, the key issue is to improve the extinction ratio. It becomes a question of reducing the modes of the device.

### 2.2. UV Writing EPO/Graphene Thermal Conduction Layer and Side Electrode

**Figure 5:**
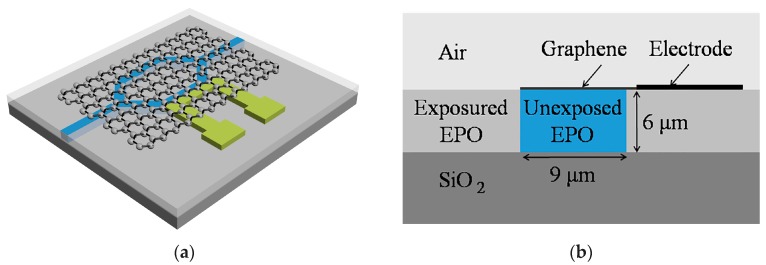
(**a**) The schematic of UV-writing thermal optical switch; (**b**) Schematic diagram of the cross-section of the device.

UV-writing method is introduced to reduce the modes in the waveguide and increase the waveguide dimension. Figure 5 shows the UV-writing device structure. M-Z structure parameters are the same with the above wet etching thermal optical switch. EPO (micro resist technology GmbH, Berlin, Germany) is selected as the polymer waveguide core material due to its high stability and low optical loss. The exposure of EPO leads to the cross link of the material. The refractive index difference between the cross linked EPO and the uncross linked EPO is calculated to be 0.001 by the ellipsometer (SPEL M-2000VI). The refractive index of exposed EPO and unexposed EPO are 1.6099 and 1.6109, respectively. The thermal conductivity is 0.2 W/(m·K). The side electrode is introduced to reduce the switching time. The electrode is designed to fabricate adjacent to the waveguide due to the low absorption of the metal at 532 nm. Two-dimensional (2D) material (such as graphene) layer is aimed to increase the heating efficiency. The graphene layer is designed to reduce the power consumption and switching time. The thermal conductor of the material is simulated with the parameter of 5300 W/(m·K).

**Figure 6:**
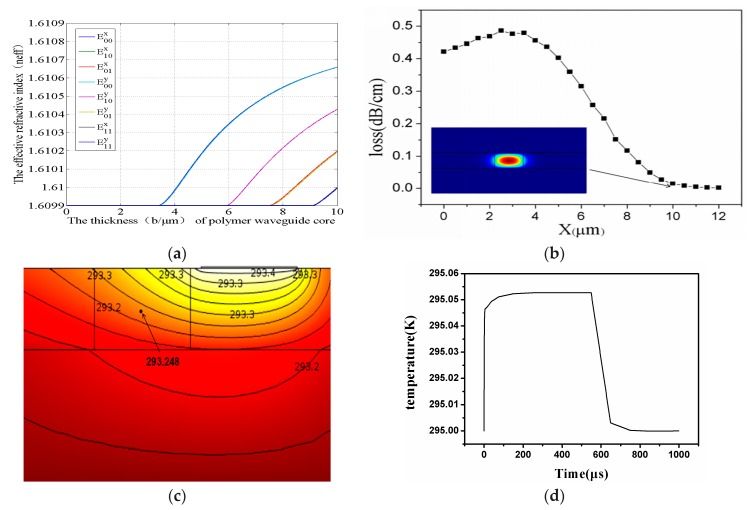
(**a**)The single mode condition of UV-writing waveguide; (**b**) The optical field and the absorption of the electrode. (**c**) The thermal field with side electrode and graphene conduction layer; and, (**d**) The switching properties of the UV-writing polymer optical switch.

Matlab calculates the relationship between the thickness and effective refractive index, as shown in Figure 6a. The exceptional loss that is caused by the side electrode is simulated in Figure 6b. With the electrode position at 1μm beside the waveguide, the absorption loss is 0.014 dB/cm. Figure 6c shows the thermal field with optimized side electrode and graphene thermal conduction layer. The UV-writing device is also simulated with the power consumption of 3.14 mW (without graphene) and 1.5 mW (with graphene). The side electrode UV-writing switching properties are calculated to be 52 μs (rise) and 89 μs (down). The switch with assisted graphene has a switching time of 18.2 μs (rise) and 85 μs (down), as shown in Figure 6d.

We compare the results of the thermal optical witches in this paper with those of other reported polymer thermal optical switches [20,21], as shown in Table 1. In Table 1, we can see that the TO switch at 532 nm would have lower power consumption, due to its short wavelength. Although the extinction ratio in this paper is lower, the low extinction ratio problem would be solved by the UV-writing technique. In addition, graphene has a relatively large thermal conductivity, which is conducive to reducing the switching response time and power consumption.

## 3. Conclusions

In this letter, a polymer-silica hybrid thermal optical switch at 532 nm was simulated and fabricated with low power consumption of 6.55 mW and the switching time of 0.23 ms (rise) and 0.28 ms (down). A UV-writing thermal optical switch with graphene layer was proposed in order to solve the low extinction ratio problem. The proposed optimized device has faster switching time (18.2 μs and 85 μs) and higher heating efficiency (power consumption of 1.5 mW). Future work will be working on the fabrication of 2D material based UV-writing thermal optical device. The device can be used in the manipulation of the visible light communication and the wearable device.

## Figures and Tables

**Table 1:** Comparisons among the performances of this thermal optical switch and those of other reported polymer thermal optical switches.

Reference	Structure (Core/Upper-Clading/Under-Cladding)	Wavelength (nm)	ER (dB)	PC (mW)	RT (μs)	FT (μs)
9	ZPUcore/ZPUcladding/ZPUcladding	1550	<−70	200	<10000	
10	SiO_2_-TiO_2_-PMMA/SiO_2_-PMMA/SiO_2_-PMMA	650	8.2	22.6	400	780
11	SU-8/PMMA/SiO_2_	1550	18.3	13	73.5	96.5
20	Doped PMMA/PMMA/PMMA	650	23.4	5.3	464.4	448.0
21	SU-8/PMMA/SiO_2_	1550	26.5	7.2	106	93
This work	SU-8/PMMA/SiO_2_	532	4.8	6.55	230	280
UV-writing with graphene	EPO/air/SiO_2_	532	-	1.5	18.2	85

^1^ IL, insertion loss; ER, extinction ratio; PC, power consumption; RT, rise time; FT, fall time.
